# Association of the triglyceride-glucose index and phlegm-dampness constitution with subclinical carotid atherosclerosis in overweight and obese individuals

**DOI:** 10.3389/fcvm.2026.1907694

**Published:** 2026-08-04

**Authors:** Zihan Qiang, Yunhan Wang, Yan Li, Yingshuai Li

**Affiliations:** Wangqi Academy, National Institute of Traditional Chinese Medicine Constitution and Preventive Medicine, Beijing University of Chinese Medicine, Beijing, China

**Keywords:** obesity, overweight, phlegm-dampness constitution, subclinical carotid atherosclerosis, TyG index

## Abstract

**Objective:**

This study aims to investigate the association between the triglyceride-glucose (TyG) index and Phlegm-dampness constitution (PDC), as well as their joint association, on subclinical carotid atherosclerosis (SCAS) in an overweight and obese population.

**Methods:**

In this cross-sectional study, 217 overweight or obese participants were enrolled. The TyG index was categorized at the median into high and low TyG groups, and a four-category joint exposure variable was developed based on PDC status. Multivariable logistic regression was employed to assess the relationship between joint exposure and SCAS, with a stratified analysis conducted by PDC.

**Results:**

SCAS was identified in 67 of 217 participants (30.9%). The TyG index (AUC 0.681) demonstrated superior performance compared to HOMA-IR (0.585) and BMI (0.515) in distinguishing SCAS. The AUC increased from 0.704 (base model: age, sex, BMI) to 0.779 after adding the TyG index (DeLong *P* = 0.005) and remained essentially unchanged after further adding PDC (0.783, DeLong *P* = 0.220). Among the four groups, the highest prevalence of SCAS was noted in the high TyG/PDC group (50.0%), while the lowest prevalence occurred in the low TyG/PDC group (12.0%). After adjusting for age, sex, and BMI, the odds of SCAS were significantly increased in the high TyG/PDC group (OR=4.83, 95% CI: 1.67–13.95). In stratified analysis, the TyG–SCAS association was numerically stronger in the PDC subgroup (OR=11.81) than in the non-PDC subgroup (OR=2.88), although the interaction was not significant (*P* = 0.252).

**Conclusion:**

The coexistence of a high TyG index and PDC was associated with markedly higher odds of SCAS. The finding that the TyG–SCAS association appeared stronger in the PDC subgroup is exploratory and requires validation in larger prospective studies.

## 1 Introduction

The prevalence of overweight and obesity in China is increasing due to lifestyle changes, leading to metabolic disorders like dyslipidemia and insulin resistance, which significantly elevate the risk of atherosclerotic cardiovascular disease (ASCVD) (1, 2). Despite similar body mass index (BMI), overweight or obese individuals exhibit varying risks of cardiovascular events, indicating limitations in risk assessment based solely on BMI (3, 4). Insulin resistance (IR) is a key factor in the progression of obesity, dyslipidemia, and vascular endothelial damage, serving as a common pathological mechanism underlying these metabolic issues. While the hyperinsulinemic-euglycemic clamp is the current gold standard for IR assessment, its invasive nature and complexity hinder its widespread clinical use (5). The triglyceride-glucose (TyG) index, a simple surrogate for IR, combines blood glucose and lipid levels and has demonstrated superior predictive performance for subclinical carotid atherosclerosis (SCAS) compared to the homeostatic model assessment of insulin resistance (HOMA-IR) in previous studies (6, 7).

Despite the TyG index's predictive value, significant variability in cardiovascular risk persists among patients with similar metabolic profiles (8). The “constitution-as-soil” theory in traditional Chinese medicine (TCM) introduces a novel perspective on this diversity, suggesting that constitution serves as the foundation for disease development and is associated with an individual's predisposition to specific illnesses (9). Phlegm-dampness constitution (PDC), characterized by a tendency toward overweight, a sticky sensation in the mouth, a thick greasy tongue coating, and a heavy sensation in the limbs, is attributed to spleen dysfunction with internal accumulation of water-dampness and phlegm turbidity, and closely parallels the metabolic syndrome profile in modern medicine (10). Epidemiological research has indicated that individuals with PDC face a heightened risk of hypertension, diabetes, and dyslipidemia compared to those with other constitution types (11–13). Nevertheless, investigations into the combined evaluation of the TyG index and PDC concerning subclinical vascular damage are limited, and whether their co-occurrence signifies an elevated risk of carotid atherosclerosis requires further exploration.

The present study focused on an overweight/obese population for analysis through a cross-sectional survey to investigate the connections between the TyG index, PDC, and their joint association with SCAS. Additionally, it examined whether the TyG-SCAS association differs by PDC status, aiming to offer insights for early identification of cardiometabolic high-risk groups using an integrated approach of TCM and Western medicine.

## 2 Materials and methods

### 2.1 Study design and participants

This cross-sectional study was conducted at Dongfang Hospital of Beijing University of Chinese Medicine and Beijing Ren'an Hospital from June 2024 to June 2025. The study protocol received approval from the Medical Ethics Committee of Beijing University of Chinese Medicine (No: 2023BZYLL1008), and written informed consent was obtained from all participants before enrollment. Participants were recruited from individuals presenting with overweight, obesity, or suspected dyslipidemia, as well as from health management screening populations. Potential participants underwent preliminary eligibility assessment (age, BMI, waist circumference, and medical history) before formal enrollment. Of 218 participants who met the initial screening criteria and provided consent, one was subsequently excluded after detailed measurement revealed that BMI and waist circumference fell below the thresholds for overweight or obesity (BMI 23.3 kg/m^2^, waist circumference 79.5 cm). The remaining 217 participants, all of whom underwent physical examination, laboratory testing, TCM constitution assessment, and carotid ultrasound examination, were included in the final analysis.

### 2.2 Eligibility criteria

Participants were eligible if they (1) were aged 18 to 65 years; (2) met the criteria for overweight or obesity (BMI ≥ 24 kg/m^2^, or waist circumference ≥ 90 cm for men and ≥ 85 cm for women) (14); (3) had completed carotid ultrasound, TCM constitution assessment, and laboratory testing with complete data; and (4) provided written informed consent. Exclusion criteria were: acute or systemic infection; pregnancy, peripartum, or lactation; malignancy, cirrhosis, or severe organ insufficiency; established clinical ASCVD or prior revascularization; severe psychiatric or cognitive disorders that would preclude participation; and missing key unverifiable variables.

### 2.3 Data collection and measurements

#### 2.3.1 Anthropometric and laboratory measurements

Body weight, height, and waist circumference were measured following standard protocols, and body mass index (BMI) was calculated as weight (kg) divided by height squared (m^2^). Blood pressure was assessed after a seated rest period of 5 min. Fasting venous blood samples were obtained following an overnight fast of at least 12 h to measure total cholesterol (TC), triglycerides (TG), high-density lipoprotein cholesterol (HDL-C), low-density lipoprotein cholesterol (LDL-C), fasting plasma glucose (FPG), glycated hemoglobin (HbA1c), fasting insulin (FINS), and parameters of liver and renal function. The homeostasis model assessment of insulin resistance (HOMA-IR) was calculated using the formula [FPG (mmol/L) × FINS (*μ*U/mL)]/22.5. FPG and TG concentrations were converted from mmol/L to mg/dL (TG:  ×  88.57; FPG:  ×  18.00) before calculating the triglyceride-glucose (TyG) index as Ln [TG (mg/dL) × FPG (mg/dL)/2] (17).   Dyslipidemia was defined as the presence of any of the following: TC ≥ 5.2 mmol/L, TG ≥ 1.7 mmol/L, LDL-C ≥ 3.4 mmol/L, or HDL-C < 1.0 mmol/L (2).

#### 2.3.2 Carotid ultrasound

Physicians with ≥ 5 years of vascular ultrasound experience conducted carotid ultrasound following a standardized protocol. Participants were examined supine with the head rotated approximately 45° to the contralateral side. A high-frequency linear-array probe was used to scan the bilateral common carotid arteries, carotid bifurcation, and proximal internal carotid arteries. Sonographers were unaware of participants’ TyG index and TCM constitution status. Carotid intima-media thickness (cIMT) was measured at end-diastole on the far wall of the distal common carotid artery and carotid sinus, with values recorded at each site. Participants were categorized into a normal cIMT group and a SCAS group based on ultrasound findings. SCAS was defined as cIMT thickening and/or carotid atherosclerotic plaque (CAP). cIMT thickening was defined as a maximum cIMT ≥ 1.0 mm at the common carotid artery or ≥ 1.2 mm at the carotid sinus. CAP was defined as a focal cIMT ≥ 1.5 mm or thickening ≥ 50% greater than the adjacent wall thickness (15).

#### 2.3.3 TCM constitution assessment

The TCM constitution was categorized following the Classification and Determination of Constitution in Chinese Medicine standard (adult version) (16). Based on a standardized 60-item questionnaire, raw and transformed scores were automatically computed to determine the dominant constitution type among the nine categories: Balanced, Phlegm-dampness, Damp-heat, Blood-stasis, Qi-stagnation, Yin-deficiency, Yang-deficiency, Qi-deficiency, and Inherited-special constitution. Constitution assessment results were used for the joint exposure analysis of the TyG index and PDC.

### 2.4 Exposure and outcome definitions

The TyG index was divided at the median into high and low groups. Participants were categorized into four combined exposure groups based on TyG group and PDC status: low TyG/non-PDC (used as a reference), low TyG/PDC, high TyG/non-PDC, and high TyG/PDC. The main outcome was SCAS, as defined above.

### 2.5 Statistical analysis

Statistical analyses were performed using SPSS 26.0. Continuous variables were presented as mean ± SD or median (IQR) and compared using t-tests or Mann–Whitney U tests. Categorical variables were presented as frequencies (%) and compared using *χ*^2^ or Fisher's exact tests. ROC curves were constructed, and AUCs were compared using the DeLong test. Logistic regression models were built by sequentially adding the TyG index and PDC to a base model (age, sex, BMI), with five-fold cross-validation. The association between the four joint-exposure groups and SCAS was assessed using multivariable logistic regression: Model 1 (unadjusted), Model 2 (adjusted for age and sex), and Model 3 (further adjusted for BMI). An interaction term (TyG group  ×  PDC) was tested, and analyses were stratified by PDC. Sensitivity analyses included using TyG tertiles and the ROC-derived optimal cutoff as alternative thresholds, further adjustment for hypertension, diabetes, smoking, and LDL-C, and exclusion of extreme laboratory values. Covariates for the primary adjusted model (age, sex, and BMI) were selected *a priori* based on their established associations with both the TyG index and carotid atherosclerosis. Additional variables — including hypertension, diabetes, smoking, and LDL-C — were reserved for sensitivity analyses because they may lie on the causal pathway between insulin resistance and atherosclerosis and their inclusion in the primary model could attenuate the estimated TyG–SCAS association. A two-sided *P* < 0.05 was considered statistically significant.

## 3 Results

### 3.1 Baseline characteristics

SCAS was identified in 67 out of 217 participants (30.9%), while the remaining 150 (69.1%) were categorized as having normal cIMT. Among the 67 participants with SCAS, 61 had isolated cIMT thickening and 6 had CAP. Given the small number of participants with plaque alone, sensitivity analyses restricted to this subgroup were not feasible due to insufficient statistical power. Table 1 displays baseline characteristics categorized by SCAS status. The SCAS group, in comparison to the normal cIMT group, exhibited significantly higher age, systolic and diastolic blood pressure, TyG index, HOMA-IR, FPG, HbA1c, TC, TG, creatinine, and dietary health scores, along with a higher prevalence of hypertension (all *P* < 0.05). The overall prevalence of dyslipidemia was 79.7% (173/217), with a higher occurrence in the SCAS group (92.5%) compared to the normal cIMT group (74.0%), showing a significant distinction in the distribution of dyslipidemia subtypes between the groups (*P* = 0.001). Mixed hyperlipidemia was the most prevalent subtype in the SCAS group (46.3%), while the normal cIMT group was mainly characterized by normal TC and TG levels (34.7%). The distribution of PDC did not differ significantly (*P* > 0.05).

**Table 1:** Baseline characteristics of participants stratified by SCAS status.

Variable	Normal cIMT (*n* = 150)	SCAS (*n* = 67)	*P* value
Demographics			
Age, years	40.00 (34.00, 44.75)	44.00 (40.00, 53.50)	**<0**.**001**
Male, *n* (%)	60 (40.0)	34 (50.7)	0.184
Obesity-related indices			
BMI (kg/m^2^)	29.55 (27.45, 32.77)	30.20 (27.45, 32.45)	0.729
Waist circumference, cm	95.8 (89.12, 103.65)	98.20 (90.40, 104.65)	0.173
Waist-to-hip ratio	0.92 (0.87, 0.97)	0.93 (0.88, 0.97)	0.073
Body fat, %	34.75 (28.90, 38.40)	33.50 (29.05, 37.25)	0.283
Visceral fat index	12.75 (11.53, 14.80)	12.60 (11.65, 14.30)	0.726
Blood pressure			
SBP, mmHg	118.00 (109.25, 127.00)	122.00 (117.50, 31.00)	**0**.**014**
DBP, mmHg	79.00 (73.00, 85.00)	83.00 (78.00, 90.00)	**0**.**008**
Glycemic and IR indices			
TyG index	8.84 (8.47, 9.27)	9.30 (8.91, 9.64)	**<0**.**001**
HOMA-IR	3.73 (2.44, 5.55)	4.05 (3.19, 5.93)	**0**.**046**
FPG, mmol/L	5.33 (4.95, 5.88)	5.52 (5.17, 6.39)	**0**.**014**
HbA1c, %	5.42 (5.16, 5.76)	5.90 (5.57, 6.45)	**<0**.**001**
Lipid profile			
TC, mmol/L	5.10 (4.59, 5.97)	5.65 (4.97, 6.33)	**0**.**005**
TG, mmol/L	1.60 (1.10, 2.24)	2.18 (1.48, 3.20)	**<0**.**001**
HDL-C, mmol/L	1.15 (0.99, 1.36)	1.18 (0.98, 1.42)	0.849
LDL-C, mmol/L	3.30 (2.78, 3.80)	3.49 (2.79, 4.04)	0.241
Dyslipidemia subtypes			
Dyslipidemia, *n* (%)	111 (74.0)	62 (92.5)	**0**.**003**
Normal TC and TG	52 (34.7)	7 (10.4)	**<0**.**001**
Isolated high TC	31 (20.7)	13 (19.4)	
Isolated high TG	29 (19.3)	16 (23.9)	
Mixed hyperlipidemia	38 (25.3)	31 (46.3)	
Hepatic, renal, and inflammatory markers			
ALT, U/L	25.33 (17.92, 43.18)	26.60 (17.36, 45.10)	0.833
Creatinine, μmol/L	64.13 (57.36, 76.15)	69.60 (60.62, 80.48)	**0**.**005**
Uric acid, μmol/L	368.50 (326.97, 436.90)	385.70 (314.05, 436.60)	0.938
hs-CRP, mg/L	2.00 (1.10, 4.17)	2.20 (1.54, 4.66)	0.105
Lifestyle			
Smoking, *n* (%)	33 (22.0)	15 (22.4)	1.000
Drinking, *n* (%)	110 (73.3)	43 (64.2)	0.228
Medical history			
Hypertension, *n* (%)	25 (16.7)	21 (31.3)	**0**.**024**
Diabetes, *n* (%)	9 (6.0)	7 (10.4)	0.380
TCM constitution			
PDC, *n* (%)	34 (22.7)	15 (22.4)	1.000
Non-PDC, *n* (%)	116 (77.3)	52 (77.6)	1.000

Bold values indicate *P* < 0.05.

### 3.2 Discriminative performance of the TyG Index and combined models

**Figure 1:**
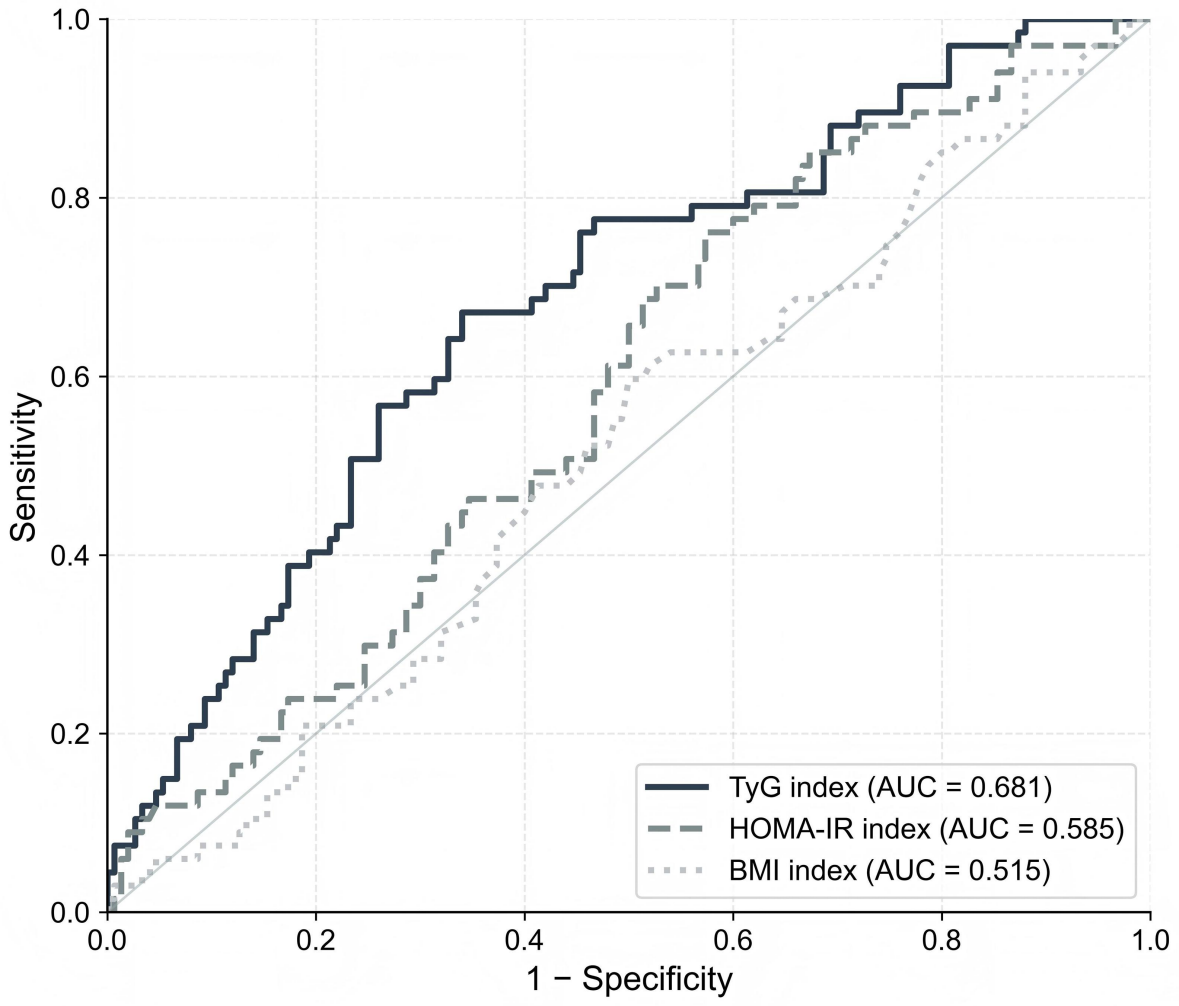
ROC curves of the TyG index, HOMA-IR, and BMI for discriminating SCAS.

ROC curve analysis (Table 2, Figure 1) revealed that the TyG index exhibited an AUC of 0.681 (95% CI: 0.619–0.743) for distinguishing SCAS, with an optimal cutoff value of 9.068 and a Youden index of 0.332, surpassing both HOMA-IR (AUC 0.585, Youden index 0.188) and BMI (AUC 0.515, Youden index 0.097). AUC comparisons using the DeLong test are summarized in Table 2. The base clinical model, including age, sex, and BMI, attained an AUC of 0.704 (95% CI: 0.643–0.765). After adding the TyG index, the AUC increased to 0.779 (95% CI: 0.724–0.834), and after further adding PDC, to 0.783 (95% CI: 0.728–0.838) (DeLong *P* = 0.220). The outcomes of five-fold cross-validation aligned with the primary analysis results.

**Table 2:** Discriminative performance of individual indicators and combined models for SCAS.

Indicator/Model	AUC (95% CI)	*P* value	Optimal cutoff	Youden index	Sensitivity (%)	Specificity (%)	CV-AUC
TyG index	0.681 (0.619-0.743)	<0.001	9.068	0.332	67.2	66.0	-
HOMA-IR	0.585 (0.520–0.650)	0.011	3.181	0.188	76.1	42.7	-
BMI	0.515 (0.448–0.581)	0.663	29.600	0.097	59.7	50.0	-
Base model	0.704 (0.643–0.765)	<0.001	-	0.321	50.7	81.3	0.700
Base + TyG	0.779 (0.724–0.834)	<0.001	-	0.484	85.1	63.3	0.761
Base + TyG + PDC	0.783 (0.728–0.838)	<0.001	-	0.486	86.6	62.0	0.750

The base model included age, sex, and BMI. The comparison of AUCs was conducted using the DeLong test, revealing the following results: TyG vs. HOMA-IR, *P* = 0.006; TyG vs. BMI, *P* < 0.001; Base + TyG vs. Base model, *P* = 0.005; Base + TyG + PDC vs. Base + TyG, *P* = 0.220. CV-AUC denotes the five-fold cross-validated AUC, while PDC represents Phlegm-dampness constitution.

### 3.3 Joint exposure and logistic regression analysis

**Figure 2:**
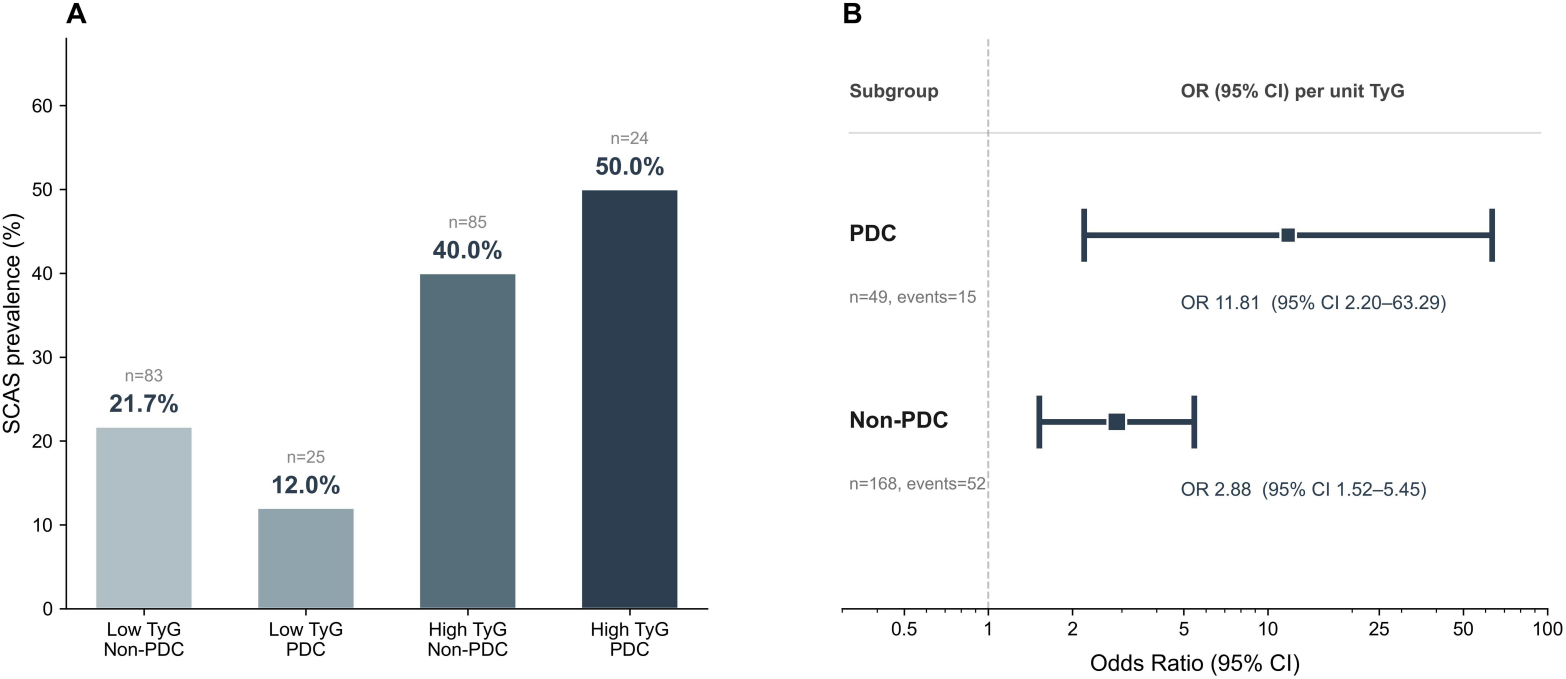
Joint association of the TyG index and PDC with SCAS. **(A)** Prevalence of SCAS across the four joint exposure groups. The TyG index was dichotomized at the median (8.955). **(B)** Stratified analysis of the association between the TyG index and SCAS by PDC status. Odds ratios are per unit increase in the TyG index, adjusted for age, sex, and BMI.

Participants were classified into four groups based on the median TyG index (8.955) and PDC status. SCAS prevalence was 21.7% in the low TyG/non-PDC group, 12.0% in the low TyG/PDC group, 40.0% in the high TyG/non-PDC group, and 50.0% in the high TyG/PDC group, with the highest prevalence observed in the high TyG/PDC group and the lowest in the low TyG/PDC group (Table 3, Figure 2A). After adjusting for age, sex, and BMI (Table 4, **Model 3**) and using the low TyG/non-PDC group as the reference, the odds of SCAS were significantly elevated in the high TyG/non-PDC group (OR 2.71, 95% CI 1.28–5.76, *P* = 0.009) and further elevated in the high TyG/PDC group (OR 4.83, 95% CI 1.67–13.95, *P* = 0.004). The low TyG/PDC group did not differ significantly from the reference (OR 0.66, 95% CI 0.17–2.63, *P* = 0.561).

**Table 3:** Prevalence of SCAS across joint exposure groups of the TyG index and PDC.

Joint exposure group	Total, n	SCAS, *n*	SCAS prevalence, %
Low TyG/Non-PDC	83	18	21.7
Low TyG/PDC	25	3	12.0
High TyG/Non-PDC	85	34	40.0
High TyG/PDC	24	12	50.0

**Table 4:** Multivariable logistic regression of the association between joint exposure groups and SCAS.

Joint exposure group	Model 1OR (95% CI)	Model 2OR (95% CI)	Model 3OR (95% CI)
Low TyG/Non-PDC	1.00 (reference)	1.00 (reference)	1.00 (reference)
Low TyG/PDC	0.49 (0.13–1.83)	0.67 (0.17–2.65)	0.66 (0.17–2.63)
High TyG/Non-PDC	2.41 (1.22–4.75)*	2.75 (1.30–5.83)**	2.71 (1.28–5.76)**
High TyG/PDC	3.61 (1.39–9.39)**	5.03 (1.77–14.34)**	4.83 (1.67–13.95)**

Model 1: unadjusted; Model 2: adjusted for age and sex; Model 3: adjusted for age, sex, and BMI.

*P < 0.05.

**P < 0.01.

### 3.4 Stratified analysis by phlegm-dampness constitution

Stratified analysis by PDC status (Figure 2B) revealed that the association between the TyG index and SCAS was approximately four times stronger in the PDC subgroup (OR 11.81, 95% CI 2.20–63.29, *P* = 0.004) than in the non-PDC subgroup (OR 2.88, 95% CI 1.52–5.45, *P* = 0.001). The wide confidence interval in the PDC subgroup reflects the limited sample size (*n* = 49, SCAS events = 15) and indicates substantial uncertainty around the point estimate. However, the interaction term was not statistically significant (P for interaction = 0.252). Accordingly, this observation should be regarded as exploratory and does not provide confirmatory evidence of effect modification.

### 3.5 Sensitivity analyses

Sensitivity analyses were conducted to evaluate the robustness of the primary findings. When the TyG index was categorized into tertiles rather than using the median-based dichotomy, participants in the highest tertile (T3) had significantly higher odds of SCAS than those in the lowest tertile (T1) (OR 4.37, 95% CI 1.93–9.91, *P* < 0.001), consistent with a dose-response gradient. After further adjustment for hypertension, diabetes, smoking, and LDL-C, the high TyG/PDC group remained significantly associated with SCAS (OR 4.12, 95% CI 1.34–12.67, *P* = 0.014). Excluding two participants with extreme laboratory values (TC > 11 mmol/L or hs-CRP > 40 mg/L) yielded consistent results (high TyG/PDC: OR 3.70, 95% CI 1.23–11.15, *P* = 0.020). Collectively, these sensitivity analyses support the robustness of the primary findings.

## 4 Discussion

In a cross-sectional analysis of 217 overweight and obese individuals, we found that the TyG index independently distinguished SCAS, surpassing both HOMA-IR and BMI, as validated by the DeLong test. Among participants with concurrent high TyG and PDC, the prevalence of SCAS rose to 50.0%, with an adjusted odds ratio of 4.83 compared to the reference group. When stratified by PDC, the association between TyG and SCAS was about four times more pronounced in the PDC subgroup than in the non-PDC subgroup. These results suggest that PDC may not act as a standalone risk factor for SCAS; the observation of a numerically stronger TyG–SCAS association in the PDC subgroup is exploratory and warrants prospective validation.

### 4.1 The TyG index outperforms conventional indices in discriminating SCAS

The TyG index, a composite measure based on fasting glucose and triglycerides, provides a comprehensive assessment of glycolipid dysregulation associated with insulin resistance. Its superior discriminatory ability compared to HOMA-IR has been corroborated by a systematic review conducted by Tao et al. (7). The absence of a significant correlation between BMI and SCAS in this study may be partly due to the limited BMI range and the generally high adiposity levels within the study cohort. This observation underscores a genuine clinical dilemma in stratifying risk among overweight and obese individuals: despite similar levels of adiposity, the extent of vascular damage can vary significantly, highlighting the inadequacy of BMI alone in effectively differentiating risk profiles (8). Blood pressure, glycemic status, and lipid measures were not included as primary covariates because they may lie on the causal pathway between insulin resistance and SCAS rather than acting as pure confounders; their inclusion in the primary model could therefore attenuate the estimated TyG–SCAS association. Sensitivity analyses further adjusting for hypertension, diabetes, smoking, and LDL-C yielded consistent results (OR 4.12, *P* = 0.014), supporting the robustness of the primary findings.

### 4.2 Phlegm-dampness constitution may identify individuals with greater TyG-associated vascular risk

In the present study, the distribution of PDC did not differ between the SCAS group and the normal cIMT group, which contrasts with the findings of Wu et al. (18), who reported that PDC was the most prevalent constitution type among 836 patients with carotid atherosclerosis. However, that study included only participants with a confirmed carotid atherosclerosis diagnosis and lacked a normal control group, which may have overestimated the apparent association between constitution and carotid atherosclerosis. By employing carotid ultrasound to objectively classify participants, the present study provides a more accurate reflection of the relationship between TCM constitution and subclinical vascular injury. A previous study indicated that cIMT was greater in hypertensive patients with PDC compared to those with a balanced constitution (19), but it similarly failed to investigate the combined effects of constitution and metabolic indicators. In the present study, SCAS prevalence differed markedly by TyG status within the PDC group (12.0% at low TyG vs. 50.0% at high TyG), suggesting a combined contribution of constitutional factors and metabolic indices. Prior work has identified alcohol consumption (20) and dyslipidemia (21) as factors that may strengthen the TyG–atherosclerosis association. In line with these findings, our stratified data raise the possibility that PDC could similarly delineate subgroups with differential TyG-associated vascular risk. However, the present study was not designed to test this hypothesis with adequate power, as reflected by the non-significant interaction term (*P* = 0.252). Whether PDC represents a clinically meaningful effect modifier of the TyG–SCAS relationship remains an open question that should be addressed in adequately powered prospective studies.

### 4.3 Joint effect of the TyG Index and phlegm-dampness constitution

The “constitution-as-soil” theory (9) suggests that an individual's constitution acts as the “soil” for disease development, with a biased constitution predisposing individuals to specific disease categories that share common pathogenic mechanisms. Our quantitative findings support this framework: the PDC “soil” does not directly lead to SCAS; however, when the “seed” of glycolipid dysregulation, indicated by a high TyG index, is present, the risk of vascular injury may be more pronounced. According to TCM theory, the spleen is responsible for the processes of transportation and transformation and serves as the origin of phlegm. Individuals who are overweight or obese tend to overconsume fatty and sweet foods, which can impair the function of the spleen. Consequently, the essence of food is not adequately transformed and distributed; instead, it accumulates as phlegm turbidity and dampness, leading to stagnation within the vessels. Over time, the congealing of phlegm and blood stasis results in the formation of intravascular masses. Zhu et al. (11) demonstrated a significant positive correlation between PDC and overweight or obesity in a large population-based sample, thereby providing epidemiological evidence for the metabolic susceptibility associated with this constitutional type.

Dyslipidemia serves as a critical biological substrate for phlegm formation, with macrophages engulfing oxidized lipoproteins to create foam cells representing a microscopic vascular manifestation of the TCM concept that “the spleen is the source of phlegm.” This pathological process parallels the overload of free fatty acids and the hepatic oversecretion of VLDL observed in states of insulin resistance. Wang et al. (10) demonstrated genomic-level associations between PDC and the expression of genes involved in lipid metabolism and inflammatory pathways, thereby reinforcing the alignment between TCM and Western medicine at the mechanistic level.

In recent years, advancements in the biological characterization of PDC have yielded molecular evidence supporting the aforementioned mechanistic understanding. In 2025, Wang Qi's team reported in Cell Discovery that individuals with PDC exhibit a significant reduction in the abundance of intestinal Flavonifractor plautii, accompanied by a decrease in its metabolite, phytosphingosine. Phytosphingosine activates the hepatic nuclear receptor PPAR*α*, which regulates the expression of genes involved in fatty acid oxidation and cholesterol metabolism (22). Fecal microbiota transplantation from PDC donors into mice—regardless of the donors’ normal metabolic profiles—induced obesity, hyperlipidemia, and insulin resistance, thereby establishing a causal role for constitution-associated gut dysbiosis in driving metabolic disorders. Subsequent research further demonstrated that gut dysbiosis in obesity-prone individuals results in reduced levels of the secondary bile acid GDCA, which impairs TGR5-mediated brown adipose thermogenesis and GLP-1 secretion (23). These findings indicate that individuals with PDC possess metabolic vulnerabilities at multiple levels, including gut microbiota, metabolites, and nuclear receptor signaling. Consequently, when the glycolipid stress represented by a high TyG index is imposed on this vulnerable background, the resulting oxidative stress and activation of inflammatory pathways may be more pronounced than in non-PDC individuals, thereby contributing to a stronger vascular effect of the TyG index.

### 4.4 Clinical implications and limitations

From a clinical translation perspective, the TyG index, requiring solely fasting glucose and triglycerides, and TCM constitution assessment, based on a standardized questionnaire, are cost-effective noninvasive tools. Incorporating the TyG index into the basic clinical model raised the AUC from 0.704 to 0.779 (DeLong *P* = 0.005), with a marginal increase to 0.783 (DeLong *P* = 0.220) upon adding PDC. This implies that PDC's individual discriminative contribution at the population level is limited, emphasizing its role in identifying heterogeneity among individuals with a high TyG index. Overweight and obese individuals with elevated TyG index and PDC may benefit from prioritized carotid ultrasound screening, intensified lifestyle intervention, and phlegm-resolving constitution regulation, aligning with the TCM principle of preventive measures pre- and post-disease onset.

Acknowledging several limitations is essential for this study. The cross-sectional design precludes causal inference. The sample size, especially in the high TyG/PDC subgroup with only 24 participants, was modest, necessitating validation of effect modification analysis in larger samples. In particular, the OR of 4.83 (95% CI 1.67–13.95) for the high TyG/PDC subgroup, although statistically significant, should be interpreted with caution given the wide confidence interval. TCM constitution assessment relies on self-reported questionnaires, potentially introducing reporting bias. Discrepancies in dietary patterns and physical activity between the PDC and non-PDC groups may exist, and residual confounding cannot be entirely ruled out. Subsequent studies should integrate objective biomarkers like metabolomics and employ multicenter, large-sample, prospective cohort designs to further corroborate the effect-modifying role of PDC and investigate its underlying molecular mechanisms.

## 5 Conclusion

In summary, the TyG index exhibited independent discriminative value for SCAS within an overweight and obese population, while traditional adiposity measures demonstrated limited utility in this population. Individuals with coexisting high TyG and PDC had markedly elevated odds of SCAS. Whether PDC modifies the vascular effects of the TyG index requires validation in adequately powered prospective studies. For overweight and obese individuals with both a high TyG index and PDC, prioritized carotid ultrasound screening may enhance precision risk stratification through the integration of metabolic indicators and TCM constitution.

## Data Availability

The raw data supporting the conclusions of this article will be made available by the authors, without undue reservation.
